# Novel oral Anticoagulants in Non-Valvular Atrial Fibrillation

**DOI:** 10.2174/187152571201141201091848

**Published:** 2014-04

**Authors:** Rose M.F.L. da Silva

**Affiliations:** Department of Internal Medicine, Faculty of Medicine, Federal University of Minas Gerais, Brazil

**Keywords:** Anticoagulants agents, apixaban, atrial fibrillation, dabigatran, rivaroxaban, systemic embolism

## Abstract

Atrial fibrillation is the most frequent arrhythmia in clinical practice, reaching 2% of the people in the world and is associated with systemic embolism. Thus, the use of anticoagulants is indicated if CHA2DS2-VASc score ≥2 or in patients with previous transient ischemic attack or stroke. For decades, warfarin, a vitamin K antagonist, was the only choice for chronic oral anticoagulation. Recently, novel oral anticoagulants (NOACs) have been introduced, offering similar (or better) effectiveness, safety, and convenience to the vitamin K antagonists. Dabigatran was the first NOAC approved and is a direct thrombin inhibitor. Rivaroxaban and apixaban are factor Xa inhibitors. They display rapid onset of action, more predictable of pharmacological profile, less interactions with other drugs, lack of significant effects in the diet, and less risk of intracranial hemorrhage than warfarin. Despite that dose adjustment is necessary for patients with chronic kidney disease or according to body weight, these new drugs do not require regular monitoring. There are recommendations for the start and follow-up therapy with NOACs, planning for cardioversion, ablation and surgical interventions and the management of bleeding. This article is a review of the major studies of the NOACs. The clinical use of these drugs in patients with non-valvular atrial fibrillation is presented.

## INTRODUCTION

Atrial fibrillation (AF) affects 2% of the people; its prevalence rises with age, reaching a rate of 15% in those aged 80. This arrhythmia is associated with poorer quality of life, intolerance to exercise, systemic embolism, hospitalization, cardiac failure and a two-fold increase in the mortality rate. There is a 5-fold higher risk of stroke, which increases with age, reaching a risk of 23.5% between 80 and 89 years ofage [1-4]. In developed countries, the majority of patients (95.8%) have non-valvular AF [5]. For prevention of systemic embolism and/or stroke, the use of anticoagulants is indicated if CHA_2_DS_2_-VASc score ≥2 or in patients with previous transient ischemic attack or stroke [1,  2]. 

Warfarin, a vitamin K antagonist, was the only choice for chronic oral anticoagulation for more than half a century. However, there are interactions with food, drugs, alcohol, liver function, as well age-related alterations and genetic variations. The use of warfarin requires periodic monitoring of the dosage of international normalized ratio (INR) and its efficacy and safety depend upon time in the therapeutic range. There is underuse of this oral anticoagulation for real-world AF patients with an elevated risk of stroke [6] and low mean time therapeutic range [7]. Recently, novel oral anticoagulants (NOACs) have been introduced, offering similar (or better) effectiveness, safety, and convenience of the vitamin K antagonists, not requiring laboratory monitoring. Ximelagatran was an oral direct thrombin inhibitor and was never approved for use due to risk of hepatotoxicity.Idraparinux is a factor Xa inhibitor subcutaneously long-acting but its use was also not approved due to the significant increase in bleeding [8]. Dabigatran, which is a direct thrombin inhibitor, and rivaroxaban and apixaban, which are factor Xa inhibitors, were NOACs approved for prevention of embolism in patients with non-valvular AF. Knowledge of the action of these novel anticoagulants, their dosage, adverse effects and interactions with other drugs is very important, given the high prevalence and prognosis of AF. 

## DABIGATRAN

Dabigatran was the first NOAC approved in many countries worldwide (in 2008 in European Union and by the Food and Drug Administration - FDA- in October 2010) for the prevention of stroke and blood clots from AF basedon the results of the RE-LY (Randomized Evaluation of Long-term Anticoagulant Therapy, Warfarin, compared with Dabigatran) trial [9-1]. This oral direct reversible thrombin inhibitor connects to thrombin with high specificity and affinity, inactivating both fibrin-bound as well as unbound thrombin. Being highly polar and lipophobic, it is not absorbed well from the gut. Dabigatran etexilate is an oral prodrug containing a tartaric acid pellet that creates an acid microenvironment that enables gut absorption. Thus, it is absorbed rapidly and hydrolyzed completely to the active molecule, dabigatran, by ubiquitous non-specific esterases in the gut, plasma, and liver. Dabigatran can be administered with or without food and its peak is within 2-3 h of oral administration. The dominant elimination pathway is renal excretion, which accounts for approximately 80%. However, dabigatran exposure is also 20–30% higher among elderly women than among elderly men, reflecting sex-related differences in creatinine clearance (CrCl) rates. Dabigatran is a substratum for the P-glycoprotein transporter and drugs that affect this system will influence its pharmacokinetics [12,  13]. Thus, the drugs that should not be co-administered are potent inhibitors of P-glycoprotein (cyclosporine, itraconazole, systemic ketoconazole, tracolimus, dronedarone), P-glycoprotein inducers (phenytoin, carbamazepine, rifampicin, St. John’s wort - *Hypericum perforatum*) and protease inhibitors. Drugs that should be co-administered with caution are other potent inhibitors of P-glycoprotein, such as verapamil, quinidine and amiodarone. Reduction of dose to 110 mg twice daily of dabigatran should be done because of potential drug interactions with verapamil, amiodarone and quinidine. Antacids and proton pump inhibitors decrease the plasma concentration of dabigatran. Therefore, dabigatran should be ingested 2 h before those medications [14]. The most adverse event associated with both dabigatran doses is dyspepsia, which is probably related to tartaric acid within the capsule [13, 14]. The pharmacodynamic and pharmacokinetic profiles of NOACs are summarized in Table **1** [12-15].

Dabigatran showed its noninferiority to warfarin for preventing stroke or systemic embolism in patients with non-valvular AF in RE-LY trial (18,113 patients, mean age 71.5 years, 63.5% male, mean CHADS_2_ 2.1) [9]. At a dose of 150 mg twice a day, it was found to be superior to warfarin at reducing the systemic or stroke (annual risk of primary endpoint was 1.1% in the dabigatran group with dose of 150 mg; *p *value < 0.001 for superiority). The rate of bleeding with dabigatran use was not greater than with warfarin use in patients using either drug for the first time. Dabigatran provoked less major hemorrhagic phenomena than warfarin (risk of 2.71% per year in the dabigatran at a dose 110 mg twice a day group and 3.36% per year in the warfarin group; p value=0.003), and that there was no significant difference in the risk of major bleeding in the 150 mg dabigatran group and the warfarin group (risk of 3.11% per year in the dabigatran group; p value=0.31). The rate of bleeding with dabigatran use was likely secondary to the underreporting of bleeding events related to warfarin use [9,  16]. 

In RE-LY trial, myocardial infarction occurred in annual rates of 0.82 % and 0.81 % with dabigatran 110 or 150 mg BID, against a rate of 0.64 % with warfarin [9]. But, in a large-scale cohort study, relative to warfarin, there was a nonsignificant tendency for lower rates of myocardial infarction with dabigatran among vitamin K antagonists (VKA) "new starters" users; however, there was a nonsignificant trend for increased occurrence of myocardial infarction among the prior VKA-experienced users. An increased myocardial infarction rate relative to warfarin among prior VKA-experienced users was clearly significant during the early follow-up period inferior of 60 days [17].

Dose of 150 mg twice a day has been approved for patients with CrCl > 30mL/min, and dose of 75mg twice daily has been approved in patients with CrCl between 15 and 30 mL/min, while dabigatran is not approved for persons with a CrCl less than 15 mL/min. Low doses may also be considered in patients aged 75 to 80 years, patients with moderately reduced kidney function and other patients who are at increased risk of bleeding. Renal function should be monitored at least once a year if their kidney function is mildly to moderately reduced or if they are over 75 years old [2, 10,  11,  18].

## RIVAROXABAN

Rivaroxaban is the second new oral anticoagulant approved in September 2008 by European Medicines Agency (EMA) and by the FDA based on the results of the ROCKET AF (Rivaroxaban Versus Warfarin in Nonvalvular Atrial Fibrillation) trial (14,264 patients, 60.3% male, median age 73 years, mean CHADS 3.47) [1,  2,  18,  19]. In a double-blind trial, the primary end point (systemic embolism or stroke) occurred at the rate of 1.7% per year in the rivaroxaban group (at a dose of 20 mg per day) and at the rate of 2.2% per year in the warfarin group (p value <0.001 for non-inferiority). Major and non-major clinically relevant hemorrhagic phenomena occurred at the rate of 14.5% per year in the warfarin group and of 14.9% per year in the rivaroxaban group, with significant reductions in fatal bleeding (0.2% vs. 0.5%, p value=0.003) and intracranial hemorrhage (0.5% vs. 0.7%, p value=0.02) in the rivaroxaban group.

Rivaroxaban is a direct factor Xa inhibitor, which connects reversibly to the active site of factor Xa, withhigh specificity, and acts independently of endogenous antithrombin. So, it suppresses the production of new molecules and plasma thrombin has no significant effect on the activity of the existing thrombin. It is rapidly absorbed and its peak is within 2-4 hours after oral administration. Rivaroxaban has a dual mode of elimination; two-thirds of the dose undergoes metabolic degradation and one thirdof the dose is eliminated as unchanged drug in the urine. Half of the dose undergoes metabolic degradation isexcreted by the kidney and half by the hepatobiliary route where is metabolized by cytochrome P450 and mechanisms CYP independent. Rivaroxaban is also a substrate of P-glycoprotein [12-15]. Thus, this drug should not be co-administered with potent inhibitors of both cytochromeP450 3A4 and P-glycoprotein (azole antimycotics and human immunodeficiency virus protease inhibitors) and dronedarone. Drugs that co-administered with caution should be strong inducers of cytochrome P450 3A4, such as rifampicin, carbamazepine and phenytoin. There is no interactionwith proton pump inhibitors. The pharmacodynamic and pharmacokinetic profiles of rivaroxaban are summarized in Table **1**.

The most common side effects (between 1 and 10 patients in 100) of rivaroxaban are anemia and bleeding [18]. A dose of 15 mg once daily is indicated for patients with a CrCl between 15 and 50 mL/min and for patients with a CrCl of >50 mL/min, the recommended dose is 20 mg per day. Rivaroxaban is not approved for use in persons with a CrCl <15 mL/min, like dabigatran.

## APIXABAN

Apixaban is the third new oral anticoagulant approvedby the FDA and by EMA in May 2011, based on the ARISTOTLE (Apixaban for Reduction in Stroke and Other Thromboembolic Events in Atrial Fibrillation) [2,  18,  20]. This was a randomized double-blinded trial comparing dose-adjusted warfarin (INR 2.0–3.0) with apixaban (5 mg bid) in 18,201 patients with atrial fibrillation and with at least one other factor for stroke. In the clinical follow-up of 1.8 years, apixaban reduced the risk of systemic embolism or strokeby 21%, major bleeding by 31%, and death by 11%. Discontinuation rates were lower with apixaban (25.3%) than with warfarin (27.5%; p value=0.001). Data from the ARISTOTLE trial that have shown apixaban is also a unique NOAC that showed a significant decrease in total mortality at the main treatment dose.

It is another oral reversible direct factor Xa antagonist. It has rapid absorption of up to 15-h half-life and the food does not affect its bioavailability. Thirty-five percent of the drug is excreted by the kidneys [12-15] (Table **1**). 

Apixaban has low potential to inhibit or induce cytochrome P450, or to form reactive metabolites, with the drug interaction potential therefore being low. Current recommendations include a reduction in dose when co-administered with drugs that are potent dual inhibitorsof cytochrome P450 3A4 and P-glycoprotein, such as ketoconazole, itraconazole, ritonavir and clarithromycin [21].

The most frequent adverse reactions are epistaxis, hematuria and bleeding in the gut and eye. The approved dose is 5 mg bid with a reduction in dose to 2.5 mg twice daily for patients with at least 80 years of age or body weight not exceeding 60 kg. The drug has not been approved for patients with severe or end stage chronic kidney disease and in patients with severe hepatic impairment [2,  18].

## OTHERS AGENTS

Edoxaban and betrixaban, other factor Xa inhibitors, are in evaluation but not yet recommended by the FDA [2]. Edoxaban was non-inferior to warfarin regarding prevention of systemic embolism or stroke and had a significant reduction of bleeding and death from cardiovascular causes in 21,105 patients with moderate to high risk AF (median follow-up, 2.8 years) [22]. Betrixaban is a new direct factor Xa inhibitor and was studied in Phase II studies in atrial fibrillation. It has different pharmacological profiles such as minimal renal clearance and minimal hepatic metabolism and a long half-life [23]. 

## COMPARISON OF THE SAFETY AND EFFICACY OF NOVEL ORAL ANTICOAGULANTS 

Although the trials for the NOACs were similar indesign and inclusion/exclusion criteria, it is hard to draw comparisons between agents to evaluate their efficacy. The NOACs have more predictable pharmacological profiles, less interactions with other drugs, lack of significant effects in the diet, and less risk of intracranial hemorrhage than warfarin. They have rapid onset of action it is not necessary to the bridge patients with parenteral and anticoagulation for initiation. The bridge may not be necessary in patients with chronic therapy who undergo invasive procedures with short interruption of anticoagulation for invasive procedures. However, temporary suspension of these agents may increase the risk of thromboembolism and, therefore, it is recommended to use another anticoagulant such as heparin. Despite dose adjustment is necessary for patients with chronic kidney disease or according to body weight, these novel agents do not require regular monitoring for INR or partial thromboplastin time [2].

Meta-analysis studies have been published on these NOACs in order to compare them [24-28]. These studies include between 44,563 and 71,683 patients with AF using oral anticoagulants, including endoxaban, demonstrating an overall clinical benefit with reduction in stroke and systemic embolism, with benefits also for women and lower rates of intracranial bleeding, but increased bleeding from the gastrointestinal tract, when compared to the use of VKA. 

There are also studies on the cost-effectiveness of NOACs, demonstrating that all were cost effective alternatives to warfarin [29-33]. There was a rapid incorporation of NOACs in clinical practice, but the female gender, low household income and higher CHADS2, CHA_2_DS_2_-VASC, and HAS-BLED scores were significantly associated with lower odds of receiving a new anticoagulant [32]. One study evaluated the cost-effectiveness of apixaban as a first-line treatment for the prevention of stroke in patients with AF eligible for VKA treatment, when compared with other NOACs, demonstrating benefits with a marginal increase in costs [33]. The incremental cost effectiveness ratios were £4497, £9611 and £5305 per quality-adjusted life-years for dabigatran 110 mg bid, dabigatran 150 mg bid and rivaroxaban 20 mg once daily, respectively. 

## PRACTICAL GUIDE ON THE USE OF NOACS

At the time of administration, fed/fasted state of the patient and other aspects of the therapy should be considered for use of NOACs. Medical knowledge and adherence to treatment are needed for successful use of these drugs in clinical practice. There are recommendations for the start and follow-up therapy with NOAC, checking the anticoagulant effect of NOACs, interactions among drugs, the management of dosing errors and hemorrhagic complications [1,  2,  34-36]. Planning for cardioversion, ablation and surgical interventions should be adopted. Baseline hemoglobin, renal and liver function should be requested. Renal function should be reassessed at least annually or when clinically indicated. CrCl should be measured using the Crockoft-Gault method.

To replace vitamin K antagonist for NOAC, the first should be discontinued and the INR closely monitored to evaluate the residual effects of the VKA. The NOAC can be started immediately when the INR approaches lower value (INR < 2.0), mainly in patients with a high risk of bleeding. INR should be monitored immediately before the next administration of the NOAC, during coadministration, and re-tested 24 hours after the last dose of NOAC. It is also recommended to monitor INR closely within the first month until stable values are achieved. If the INR is in the range of 2.0-2.5, NOAC should be started the next day. For patients with an INR > 2.5, the INR value and the half-life of the AVK to be considered to estimate the time when INR will drop below 2.5. A loading dose for acenocumarol and warfarin (elimination half-life of 40-70 hours and 3-10 hours, respectively), it is not recommended but is appropriate with phenoprocoumon (elimination half-life of 4-6 days). The interactions between the drugs and the precautions have already been discussed previously in this text.

If the patient forgot one dose, this dose may be ingested at half of the dosing interval (e.g., up to 12 h for a once daily dosing). If this is not possible, the dose should not be administered and only the next dose should be taken. In the case a double dose was taken by mistake, patient may choose not to take the next scheduled dose.

### Special Considerations for Management of NOAC Concerning the Procedures

There are special considerations regarding the use of NOAC for some procedures such as cardioversion, ablation and surgical interventions.

Since there is no test available for clotting NOACs for providing information about effective anticoagulation over the last 3 weeks prior to cardioversion, it is mandatory to verify compliance to treatment by patients. However, a prior transesophageal echocardiogram should be considered if there is doubt about the regular use of anticoagulant [34].

Using data from the ARISTOTLE with a total of 743 cardioversions that were performed in 540 patients, a recent study demonstrated that major cardiovascular events after cardioversion of AF are uncommon and the results are similar between apixaban and warfarin [37]. There are limitations in the study as the low rate of expected events after cardioversion, beyond the fact that the authors could not be sure that all recorded cardioversions were actually performed for AF. Available data suggest that apixaban (or other NOAC) should not be less effective than warfarin for this purpose, but the available data do not provide definitive responses; thus, additional research in this area is necessary [38].

With regard to ablation, to prevent thromboembolism, there is consensus that anticoagulation is indicated around the time of catheter radiofrequency ablation. Additionally, both intraprocedural heparin and oral anticoagulation for≥ 2 months postprocedure are recommended [1,  2,  34]. For patients taking dabigatran, a minimally interrupted strategy may be employed. This strategy is the suspension of dabigatran 12-24 hours before the procedure and its resumption within 4-8 hours after the procedure. For patients taking rivaroxaban, small studies with maintaining the drug demonstrated efficacy and safety results similar to those with uninterrupted use of warfarin. The use of a strategy minimally interrupted or uninterrupted with NOACs may be similar to warfarin uninterrupted strategy results, but further tests are required [39].

For patients planning to undergo surgical intervention, it should be performed 18-24 hours after the last ingestion of NOAC. For patients with renal function who will undergo procedures with a lower risk of bleeding, NOACs should be discontinued 24 h before the scheduled procedure. For procedures with greater risk of bleeding, NOAC should be discontinued 48 h earlier. NOAC can be taken 6-8 hours after the procedure [34]. In patients with CrCl < 50 mL/min, dabigatran should be withdrawn at least 3 days before surgery and apixaban should be stopped 48 h before surgery or invasive procedures, for patients with moderate to high risk of bleeding [35]. If an emergency intervention is required, the NOAC should be withdrawn. If possible, surgery or intervention should be delayed until at least 12 h and ideally 24 h after the last dose.

For management of anticoagulation around pacemaker and defibrillator surgery, in patients with an annual risk of arterial thromboembolic events > 5%, the first dose dabigatran may be taken 24 hours after surgery. In patients with a lower risk of systemic embolism (< 5%), expert opinion has recommended temporary interruption around device surgery with the suggested period of interruption varying between 3-7 days. It is recommended to restart after more than 48 hours from the intervention. More dataare needed to refine all these recommendations on the management of NOAC around the surgery [40].

### Acute Management of Bleeding

Chronic kidney disease is associated with increasedrisk of hemorrhage during therapy with dabigatran. For rivaroxaban, less fatal bleeding and less intracranial hemorrhage had been observed. And patients treated with apixaban had significantly fewer intracranial hemorrhages. Despite low rates of bleeding following the use of NOACs, these rates can reach up to 3.6% per year [41]. The probable causes of low rates of bleeding, especially intracranial, may be the action of the NOACs in the single site in coagulation cascade and no direct effect on factor VIIa. 

Measurement of NOAC concentration and/or activity in plasma can be helpful in this situation. The NOACs increase or do not change the prothrombin time. Dabigatran may result in increased activated partial thromboplastin(aPTT) and rivaroxaban and apixaban may increase ornot change that test. Dabigatran also prolongs thrombin clotting time. There are coagulation tests with specific calibrators or standards that provide accurate quantitative estimates of anticoagulant plasma concentrations. For dabigatran there is the *Hemoclot* direct thrombin inhibitor assay, a dilute thrombin time performed with internal dabigatran calibrators, and for rivaroxaban and apixaban, there are anti-factor Xa assays. For anticoagulant plasma concentrations, the gold standard for dabigatran is liquid chromatography-tandem mass spectrometry [42]. Despite this, conventional coagulation tests have limitations when used to measure the effect of NOAC. 

If bleeding occurs, the anticoagulant agent should be discontinued. General measures include hemostasis, hydration, maintenance of diuresis, transfusing blood products, and waiting for the anticoagulant to be metabolised and excreted. Activated charcoal may be used to reduce the absorption of NOAC. There is an option of dabigatran for dialysis. However, rivaroxaban and apixaban are not dialyzable [2, 34,  41]. There is a reduction of the absorption of dabigatran and rivaroxaban with the administration of activated charcoal within 2-8 h of ingestion, respectively; however, there are no *in vivo* studies on this matter [35].

The administration of prothrombin complex concentrate 25 U/kg can be made in a patient with life-threatening bleeding and may be repeated once or twice. Other strategies for the management of bleeding were evaluated, including the use of recombinant factor VIIa, but there are no data about additional benefit with activated factor VII [41]. Thus, agents of reversion to the NOACs are currently not available. However, clinical trials were conducted to evaluate the efficacy and safety of an antidote to dabigatran (a Dabi-Fab), which is a humanized antibody (Fab) fragment that shares some structural features with thrombin, competitively inhibiting binding of thrombin to dabigatran. Also r-Antidote is in development, a recombinant, hemostatically inactive, protein variant of factor Xa, which competes withnative factor Xa for factor Xa inhibitors and reverses the anticoagulant effects of rivaroxaban and apixaban [42]. Desmopressin and antifibrinolytic agents like tranexamic acid and 1-aminocaproic acid can be used as adjunctive therapies in circumstances of severe bleeding. However, the mainstays of treatment are supportive measures and prompt consideration of hemostatic intervention [41].

## CONFLICT OF INTEREST

The author(s) confirm that this article content has no conflict of interest.

## Figures and Tables

**Table 1:** Pharmacodynamic and pharmacokinetic characteristics of the NOACs

Drugs Characteristics	Dabigatran	Rivaroxaban	Apixaban
Mechanism of action	Direct thrombin (factor IIa) inhibition	Director factor Xa inhibitor	Director factor Xa inhibitor
Oral bioavailability, %	6	> 80	45
Plasma protein blinding, %	35	> 90	87
Time do peak levels, hours	3	2-4	1-3
Half-life, hours	12-17	5-12	9-15
Excretion	80% renal	33% renal, 66% liver	35% renal, 75% fecal
